# Dynamic Mechanical Analysis, Morphology, Physico-Mechanical, and Performance Properties of EPDM/NBR Rubber Blends Containing Chlorosulfonated Polyethylene as a Compatibilizer

**DOI:** 10.3390/polym18010103

**Published:** 2025-12-30

**Authors:** Evgeniy Egorov, Rakhymzhan Turmanov, Rakhmetulla Zhapparbergenov, Aslan Oryngaliyev, Nurgali Akylbekov, Nurbol Appazov, Anton Loshachenko, Nikita Glukhoedov, Abdirakym Nakyp, Nadezhda Semenova

**Affiliations:** 1 Department of Physical Chemistry and Macromolecular Compounds, Faculty of Chemistry and Pharmaceutics, Chuvash State University Named after I.N. Ulyanov, 15 Moskovsky Prosp., 428015 Cheboksary, Russia; 2 Laboratory of Engineering Profile “Physical and Chemical Methods of Analysis”, Korkyt Ata Kyzylorda University, Aiteke bie Str. 29A, 120014 Kyzylorda, Kazakhstan; r.zhapparbergenov@korkyt.kz (R.Z.); nurgali@korkyt.kz (N.A.); nurasar.82@korkyt.kz (N.A.); 3 “CT Solutions” LLP, Medeu District, Ibragimova Street 9, Block B, Office 323, 050032 Almaty, Kazakhstan; a.oryngaliyev@ctsolutions.kz; 4 Interdisciplinary Resource Centre for Nanotechnology, St. Petersburg State University, 7/9 Universitetskaya Nab., 199034 St. Petersburg, Russia; a.loshachenko@spbu.ru (A.L.); n-glukhoyedov@rambler.ru (N.G.); 5 Department of Synthetic Rubber Technology, Institute of Polymers, Kazan National Research Technological University, 68 K. Marx Str., 420015 Kazan, Russia; 6 Cheboksary Production Association Named after V.I. Chapaev, 1 St. Socialist, 428038 Cheboksary, Russia; hope325@inbox.ru

**Keywords:** rubber blends, compatibilizer, chlorosulfonated polyethylene, dynamic mechanical analysis, morphology, physico-mechanical properties, performance properties

## Abstract

The article studies the influence of chlorosulfonated polyethylene CSM 40 as a compatibilizer on the curing characteristics of the rubber compound, dynamic mechanical analysis, morphology, physico-mechanical and performance properties of vulcanized rubber based on a compound of ethylene propylene diene monomer EPDM S 501A and nitrile butadiene NBR 2645 rubbers. DMA studies indicate that the temperature dependence of *tan*δ for vulcanizates with and without a compatibilizer based on EPDM S 501A/NBR 2645 at a ratio of 75/25 parts per hundred parts of rubber (phr) has a bimodal character, which indicates the incompatibility of the rubber phases. The temperature dependence for EPDM S 501A/NBR 2645 vulcanizates (25/75 phr) with and without a compatibilizer has a monomodal form, which characterizes the improved compatibility of the rubber phases. SEM showed that a clearly defined microporous structure is observed on a cleavage of vulcanizate sample EPDM/NBR (25/75 phr) without a compatibilizer; with the addition of CSM 40, this feature is retained, but becomes less pronounced. It is shown that vulcanizates containing the compatibilizer CSM 40 are characterized by increased strength properties and hardness compared to vulcanized rubber without a compatibilizer. It was established that the vulcanized rubber based on EPDM S 501A/NBR 2645/CSM 40 (25/75/5 phr) is characterized by the smallest changes in the elastic-strength properties and hardness of vulcanizates after a day of thermo-oxidative aging in air and their weight after exposure to industrial oil I-20A and standard petroleum fluid SZhR-1 at room temperature among vulcanizates based on EPDM S 501A and NBR 2645. The vulcanizate of the rubber compound, including a compound of EPDM/NBR (25/75 phr) with a compatibilizer CSM 40 in an amount of 5 phr (2.88 wt.%), is characterized by stable physico-mechanical properties and improved performance properties. This rubber compound can be used for the manufacture of rubber products operating under the influence of oils and hydrocarbon environments.

## 1. Introduction

Ethylene propylene diene monomer rubber (EPDM) is a terpolymer of ethylene, propylene, and a small amount of non-conjugated diene [1,2,3,4,5,6,7]. In the production of EPDM, ethylidene norbornene, dicyclopentadiene, or 1,4-hexadiene are usually used as the diene [2,5]. This rubber is characterized by balanced heat resistance and elasticity, especially at very low temperatures [8,9], has good resistance to ozone oxidation [10,11,12,13,14], and is characterized by excellent dielectric properties.

The fields of application of EPDM are very diverse. In the total mass of consumed products obtained from these rubbers, the largest share falls on the automotive industry. EPDM can be used in all rubber technical parts of cars, with the exception of tires and oil-resistant products. In the automotive industry, EPDM is used for the production of seals for windows, doors, trunks, hoods, brake device parts [15,16,17,18], soundproofing, shock-absorbing and anti-vibration parts [19,20,21], seat headrests [22,23], mats [24,25]. In electrical engineering and cable industries, EPDM is used for the production of cable sheaths for low, medium, high and even ultra-high voltage due to its excellent electrical insulation [26]; electrical insulating materials [27,28,29]; fittings and parts of electrical wiring (sockets, plugs, couplings, etc.) [30]. In construction, EPDM is widely used for the production of sealing profiles [31]; heat- [32,33] and sound-insulating [34,35] and water-resistant [36] coatings; roofing materials [37,38].

However, it should be noted that EPDM is not resistant to most oils, gasoline, kerosene, aromatic and aliphatic hydrocarbons, and halogenated solvents. Blending EPDM with nitrile butadiene rubber (NBR) can eliminate the above-mentioned disadvantages of EPDM, since NBR has high resistance to swelling in oils and solvents [39,40,41,42,43,44,45]. In [8], it shows that when NBR is dispersed in an EPDM matrix, the dispersion region is larger, the particle size distribution is very wide, and the interfacial adhesion is very weak, which leads to low physico-mechanical properties of vulcanizates based on a blend of NBR/EPDM rubbers. This is explained by the fact that EPDM and NBR are thermodynamically incompatible and have different vulcanization rates. Therefore, to improve the compatibility of rubbers, functional additives, so-called compatibilizers, are used [46,47,48,49,50].

Rubber compatibility is of interest from both a technical and scientific perspective. Combinations of different rubbers can yield improved properties in finished rubber products compared to individual polymers. The industrial use of rubber blends is widespread. Many rubbers, differing in chemical structure, are blended to improve the physico-mechanical properties of vulcanizates and the durability of finished rubber products [51,52]. Polymer compatibility, particularly of rubbers, can be achieved either by reducing interfacial energy, by stabilizing the polymers to prevent phase separation after blending, or by improving interfacial or interdomain adhesion [52].

Commercially useful for compatibilizing rubber blends is the polymer–polymer combination due to intermolecular forces such as van der Waals forces or dipole moments, which provide sufficient thermodynamic compatibility and prevent polymer phase separation [53,54]. Several studies have been devoted to the study of the compatibility of EPDM with other diene rubbers, in particular, NBR. For example, in [55], the compatibility of blend of natural rubber NR (62.5 wt.%), reinforced with phlogopite filler, and ethylene propylene diene rubber EPDM (37.5 wt.%) was studied using an aminosilane compatibilizer. It was shown that the combination of aminoethylaminopropyltrimethoxysilane and stearic acid ensures the compatibility of the NR/EPDM/phlogopite composite, which additionally improves the technological (rheometric) properties of the rubber compound and the elongation at break of the vulcanizates.

Thus, in the work of Pandey et al. [56], the compatibility of immiscible binary blends of NBR and EPDM was studied at their mass ratio of 50:50 phr. It was shown that the compatibility of these rubbers was achieved by adding either chlorinated polyethylene (CM) or chlorosulfonated polyethylene (CSM) as compatibilizers in an amount of 5.0 phr of each. Researchers Azizli et al. [57] examined the effect of the product of graft copolymerization of EPDM with maleic anhydride EPDM-g-MA as a compatibilizer on the compatibility of a blend of carboxylated butadiene-nitrile rubber XNBR and EPDM in the presence of organomodified montmorillonite Cloisite 15A in quantities of 0, 2.5, 5.0, 7.5 and 10.0 phr with a sulfur vulcanizing system. It was shown that the scorch time and optimum cure time decrease with increasing organoclay content Cloisite 15A, while the optimum cure time increases with increasing EPDM content in the rubber compound. Dynamic mechanical analysis (DMA) and scanning electron microscopy (SEM) revealed that the addition of the EPDM-g-MA compatibilizer at 5.0 phr ensures good compatibility of the polar phase of XNBR and the non-polar phase of EPDM at a ratio of 80:20 phr, respectively. It was found that rubber based on XNBR/EPDM (80/20) exhibits optimal elastic strength properties, thermal stability, and ozone resistance.

Botros and Tawfic [58] used maleated ethylene propylene diene rubber MAH-g-EPDM (a product of graft copolymerization of EPDM with maleic anhydride) as a compatibilizer for EPDM/NBR blends in different ratios with a sulfur vulcanizing system. Intrinsic viscosity measurements and scanning electron microscopy (SEM) showed that MAH-g-EPDM in an amount of 10.0 phr improves the compatibility and morphology of the EPDM/NBR blend. It was found that of all the component ratios studied, the vulcanizate based on the EPDM/NBR (25/75) blend compatibilized with MAH-g-EPDM has the highest elastic-strength properties, as well as good thermal and UV resistance.

In [59], it shows that the use of sulfur alone as a vulcanizing agent for a binary NR/EPDM blend did not provide satisfactory physico-mechanical properties of the vulcanizates and it was demonstrated that during the vulcanization process, sulfur is mainly in the NR phase and, as a consequence, the EPDM phase remains insufficiently vulcanized. Peroxide vulcanization provides a more uniform distribution of cross-links compared to sulfur, which has a positive effect on the physico-mechanical and performance properties of vulcanized rubbers. In this regard, it is of interest to study the effect of chlorosulfonated polyethylene CSM 40 as a compatibilizer on the curing properties of the rubber compound, dynamic mechanical analysis, morphology, physico-mechanical and performance properties of vulcanized rubber based on a blend of ethylene propylene diene monomer EPDM S 501A and butadiene-nitrile NBR 2645 rubbers at their various ratios with the peroxide vulcanization system.

## 2. Materials and Methods

### 2.1. Materials

The following rubbers and ingredients served as the basis for the rubber compound under study: ethylene propylene diene monomer rubber EPDM Suprene 501A with a Mooney viscosity ML_1+4_ (100 °C) of 46 with an ethylene content of 53.0 wt.% and ethylidene norbornene (ENB) of 4.1 wt.% (SK Global Chemical Co., Ltd., Seoul, Republic of Korea); nitrile butadiene rubber NBR 2645 with a mass fraction of acrylonitrile of 27–30%, Mooney viscosity ML_1+4_ (100 °C) of 45 ± 3 (Krasnoyarsk Synthetic Rubber Plant, Krasnoyarsk, Russia); compatibilizer—chlorosulfonated polyethylene CSM 40 with a Mooney viscosity ML_1+4_ (100 °C) of 41–50, with a chlorine content of 36.2 wt.% and sulfur of 1.07 wt.% (Haihang Industry Co., Ltd., Jinan, China); curing agent—Luperox F 40P E (1,3 1,4-bis-(*tert*-butylperoxyisopropyl)benzene) with a peroxide content of 40.0 wt.% and active oxygen of 3.59–3.97 wt.% (Seki Arkema Co., Ltd., Chilseo-myeon Haman, Republic of Korea); peroxide vulcanization coagent—triallyl cyanurate TAC 70 (powdery 70% preparation of triallyl cyanurate on finely divided silica as carrier material) with a density at 20 °C of 1.21 g/cm^3^ (Kettlitz-Chemie GmbH & Co Kg, Rennertshofen, Germany); vulcanization activators—zinc oxide (ZnO) (Empils-zinc, Rostov-on-Don, Russia), stearic acid (RossPolimer, Moscow, Russia); antioxidants—antioxidant 445 (4,4-bis(α,α-dimethylbenzyl)diphenylamine) with a melting point of 98–105 °C (Nanjing Capatue Chemical Co., Ltd., Nanjing, China), nickel dibutyldithiocarbamate, which is a non-dusting green powder with a melting point of 85–90 °C, a nickel mass fraction of 12.3–13.0% (Beraton-Rus, Perm, Russia), wax ZV-P (KhimAvangard, Dzerzhinsk, Russia); fillers—carbon black P 324 (Nizhnekamsktekhuglerod, Nizhnekamsk, Russia), carbon black P 514 (Ivanovo carbon black and rubber JSC, Ivanovo, Russia) with a specific surface area of 104–118 and 50–57 m^2^/g, respectively; softener—naphthenic oil NMR-12 with a kinematic viscosity of 10–13.5 cSt at 50 °C, a pour point of −40 °C, a flash point of 155 °C (PAN Company, Perm, Russia).

### 2.2. Preparation of Rubber Blends

To measure the intrinsic viscosity of EPDM-NBR blend solutions, various EPDM/NBR compositions (100/0, 75/25, 50/50, 25/75, and 0/100 phr) with different amounts of CSM 40 compatibilizer (0, 5.0, 7.5, and 10.0 phr) were prepared. Each blend was prepared by plasticizing the rubber blends on LB 320 160/160 laboratory rollers (Polimermash group, St. Petersburg, Russia) with a friction ratio of 1:1.25 at a roll surface temperature of 70 ± 5 °C for 5 min.

### 2.3. Preparation of the Samples

The rubber compound with and without a compatibilizer was prepared on laboratory rollers LB 320 160/160 with a friction of 1:1.25 at a roller surface temperature of 60 ± 5 °C. First, the rubber and rubber blends were plasticized for 3 min (with the addition of the compatibilizer for 2 min); then ZnO, stearic acid, antioxidant 445, nickel dibutyldithiocarbamate, wax ZV-P, ½ part of carbon black P 324 were added and mixed for 8 min (in the presence of the compatibilizer for 7 min); then the remaining ½ part of carbon black P 324, carbon black P 514, naphthenic oil NMR-12 were added and mixed for 6 min. Then triallyl cyanurate TAC 70 and Luperox F 40P E were added and mixed for 2 min.

Standard samples of all rubber compound variants were vulcanized for 30 min at 150 °C and 17.6 MPa in a P-V-100-3RT-2-PCD vulcanization press (Pan Stone Hydraulic Industries Co., Taichung City, Taiwan) to determine the physico-mechanical properties. Vulcanizate samples were thermostatic at 160 °C for 3 h in an SM 50/300-250 ShS thermostat (SM Climate, St. Petersburg, Russia).

### 2.4. Methods

#### 2.4.1. Viscosity Measurement

The prepared EPDM/NBR blends with and without a compatibilizer were dissolved in toluene to obtain solutions with different concentrations (0.6, 0.5, 0.4, 0.3, 0.2, and 0.1 g/dL). The viscosity of the EPDM/NBR blend solutions was measured using an Ostwald viscometer (capillary diameter 0.56 mm) at a temperature of 25 ± 1 °C.

#### 2.4.2. Curing Characteristics

The curing properties of the rubber compound were studied on an MDR 3000 Basic rheometer (Mon Tech Co., Buchen, Germany) at 150 °C for 30 min in accordance with ASTM D5289. Due to the marching modulus characteristics of the curves, the maximum torque value in this study is defined as the torque value at the end of the test period (30 min).

#### 2.4.3. Determination of the Crosslink Density

Crosslink density was determined using equilibrium swelling data for the vulcanizates. For this purpose, rectangular samples of vulcanizates with dimensions of 25 × 7 × 2 mm were cut out from the central part of the vulcanized rubber plate and immersed in toluene in a closed container for 72 h at room temperature in a dark place. After reaching the equilibrium degree of swelling, the sample was removed from the solvent (toluene), quickly dried with filter paper, and weighed. The swollen samples were then placed in an ES-4620 drying oven (Ekroskhim, St. Petersburg, Russia) and dried at 60 °C for 24 h to remove the solvent. The dried vulcanizate samples were weighed.

Between the average molecular weight of a segment of the molecular chain (M_c_) enclosed between two cross-links and the volume fraction of rubber in the swollen vulcanizate ($\nu_{r}$) there is the following relationship, described by the Flory-Rehner Equation (1) [60]:

(1) $$M_{c}=-\frac{\rho_{r}V_{s}(\sqrt[{3}]{\nu_{r}}-\frac{\nu_{r}}{2})}{\ln\left(1-\nu_{r}\right)+\nu_{r}+\chi \nu_{r}^{2}}$$

where χ describes the Flory-Huggins polymer-solvent interaction parameter, V_s_ is the molar volume of the solvent used (i.e., 106.27 cm^3^/mol for toluene [61]), and $\rho_{r}$ (g/cm^3^) is the density of rubber.

$\nu_{r}$ is determined by Equation (2):

(2) $$\nu_{r}=\frac{1}{\left[1+\left(\frac{W_{s}\;-\;W_{d}}{W_{d}}\right)\right]\frac{\rho_{r}}{\rho_{s}}}$$

where W_s_ is the weight of the swollen sample, W_d_ is the weight of the sample dried after swelling, $\rho_{r}$ and $\rho_{s}$ represent the density of the rubber and solvent, respectively [62,63]. ρ(EPDM S 501A) = 0.86 g/cm^3^; ρ(NBR 2645) = 0.96 g/cm^3^; ρ(CSM 40) = 1.12 g/cm^3^. Using the additivity rule, we obtain the densities for the EPDM S 501A—NBR 2645 and EPDM S 501A—NBR 2645—CSM 40 blends at different mass ratios. The solvent density ρ(toluene) = 0.8669 g/cm^3^.

The Flory-Huggins interaction parameter χ for rubbers is: χ(EPDM S 501A) = 0.496 [64,65]; χ(NBR 2645) = 0.414 [66]; χ(CSM 40) = 0.401 [67,68,69]. Applying the additivity rule, we obtain the values χ for the EPDM S 501A–NBR 2645 and EPDM S 501A–NBR 2645–CSM 40 blends at their different mass ratios.

The crosslink density $\nu_{c}$ (mol/cm^3^) is calculated using Equation (3) [70]:

(3) $$\nu_{c}=\frac{\rho_{r}}{2M_{c}}$$

#### 2.4.4. Dynamic Mechanical Properties

The dynamic parameters (storage modulus, loss factor) of vulcanizates (samples 12 mm long, 4 mm wide and 2 mm thick) were studied using a Netzsch DMA 242 D Artemis dynamic mechanical analyzer (NETZSCH Group, Selb, Germany) in the temperature range from −90 to 90 °C at a heating rate of 2 °C/min, an oscillation frequency of 10 Hz, an amplitude of 30 μm in tensile deformation mode.

#### 2.4.5. Scanning Electron Microscopy (SEM)

Rubber Blends micromorphology both planar and cross-section geometry, was studied on a Carl Zeiss Auriga Laser (Carl Zeiss SMT, Oberkochen, Germany) cross beam work station under 20 keV. Cross-sections were prepared by submerging the sample in liquid nitrogen and fracturing.

#### 2.4.6. Physico-Mechanical Properties

Tensile strength and elongation at break of vulcanizates (samples in the amount of five double-sided blades with a thickness of (2.0 ± 0.2) mm for each version of vulcanizate) were determined in accordance with ASTM D412 using a tensile testing machine REM-5-M-1-2 (Metrotest, Moscow, Russia) at a gripper speed of 500 mm/min. The mean of these indicators of the five tested samples for each variant of the vulcanizate was taken as the test result.

Shore A hardness was determined using a TVR-A hardness tester (Vostok-7, Moscow, Russia) on one sample in the form of a washer with parallel planes with a diameter of 50 mm and a thickness of 8 mm for each version of the vulcanizate according to ASTM D2240-15. Hardness was measured at three points in different places of the sample. The test result was taken as the arithmetic mean of all measurements, rounded up to a whole number.

The tear resistance was determined on five arcuate samples with a thickness of (2.0 ± 0.2) mm for each variant of the vulcanizate in accordance with ASTM D624 using an REM-5-M-1-2 tensile testing machine at a gripper speed of 500 mm/min. The arithmetic mean of five tested samples for each variant of the vulcanizate was taken as the test result.

Compression set in air was determined on three samples in the form of cylinders with a diameter and height of (9.8 ± 0.1) mm for each vulcanizate according to ASTM D395-18. The test result was taken as the arithmetic mean of three tested samples for each version of the vulcanizate.

Rebound elasticity was determined using a Shoba-type UMR-1 resilience tester (Polimermash, St. Petersburg, Russia) on two parallel-plane washer specimens with a diameter of 50 mm and a thickness of 6 mm for each vulcanizate variant, according to ASTM D2632. For each of the two specimens, the mean value of three measurements (the median) was selected. The arithmetic means of the two selected values, rounded to the nearest whole number, were taken as the test result for each vulcanizate variant.

#### 2.4.7. Performance Properties

The change in the tensile strength, elongation at break, and hardness of vulcanizates after thermal aging in air was determined according to Russian State Standard GOST 9.024-74 “Unified system of corrosion and aging protection. Rubbers. Methods of heat aging stability determination”; the change in mass after exposure to aggressive environments was determined according to Russian State Standard GOST 9.030-74 (method A) “Unified system of corrosion and aging protection. Vulcanized rubbers. Method of testing resistance to attack by corrosive media in limp state”.

The essence of the method for testing the resistance of vulcanizates to thermal aging in air is that undeformed vulcanized rubber samples were exposed to air at an elevated temperature (100 °C) in an SM 50/300-250 ShS thermostat (LLC “SPM Climate”, St. Petersburg, Russia) for 24 h and the ability of vulcanized rubbers to resist their effects is determined by changes in the following parameters: tensile strength, elongation at break and Shore A hardness. Tensile strength and elongation at break of vulcanizates before and after aging in air were determined on five samples in the form of double-sided blades with a thickness of (2.0 ± 0.2) mm. The arithmetic mean of these parameters for the five tested samples for each version of vulcanizate before and after aging in air was taken as the test result. The change in tensile strength Δ*f_p_* (%) and elongation at break Δ*ε_p_* (%) were calculated using Equation (4) and (5), respectively:

(4) $$∆f_{p}=\frac{f_{p1}-f_{p}}{f_{p}}\times 100\%$$

where *f_p_* is the value of the tensile strength before aging; *f_p_*_1_ is the value of the tensile strength after aging in air.

(5) $$∆\varepsilon_{p}=\frac{\varepsilon_{p1}-\varepsilon_{p}}{\varepsilon_{p}}\times 100\%$$

where *ε_p_* is the value of elongation at break before aging; *ε_p_*_1_ is the value of elongation at break after aging in air.

Hardness before and after thermal aging in air was measured at three different points on the same sample, a 50 mm diameter, 8 mm thick parallel-plane washer for each vulcanizate variant. Hardness change ∆*H* (Shore A units) was calculated using Equation (6):

(6) $$∆H=H_{1}-H$$

where *H* is the hardness value before aging; *H*_1_ is the hardness value after aging in air.

The essence of the method for testing the resistance of vulcanizates to the effects of liquid aggressive environments is that vulcanized rubber samples in a stress-free state were exposed to the effects of liquid aggressive environments (standard petroleum fluid SZhR-1 with an aniline point of (124 ± 1) °C, kinematic viscosity at 98.9 °C (20 ± 1) mm^2^/s and industrial oil I-20A with a density at 20 °C of 0.89 g/cm^3^, kinematic viscosity at 40 °C of 29–35 mm^2^/s) at a temperature of 23 °C for 24 h and their resistance to the specified effect is determined by the change in mass. The mass of the vulcanizate samples before and after exposure to the specified aggressive environments was determined on three rectangular samples with dimensions of 25 × 20 mm and a thickness of (2.0 ± 0.2) mm. The test result was the arithmetic mean of three tested samples for each vulcanizate variant before and after exposure to the specified liquid aggressive environments. The mass change ∆*m* (%) of the vulcanizates was calculated using Formula (7):

(7) $$∆m=\frac{m-m_{0}}{m_{0}}\times 100\%$$

where *m*_0_ is the mass of the vulcanizate sample before exposure to aggressive environments; *m* is the mass of the vulcanizate sample after exposure to aggressive environments.

## 3. Results and Discussions

### 3.1. Determining the Optimal Amount of Compatibilizer and Rubber Compound Options

**Figure 1:**
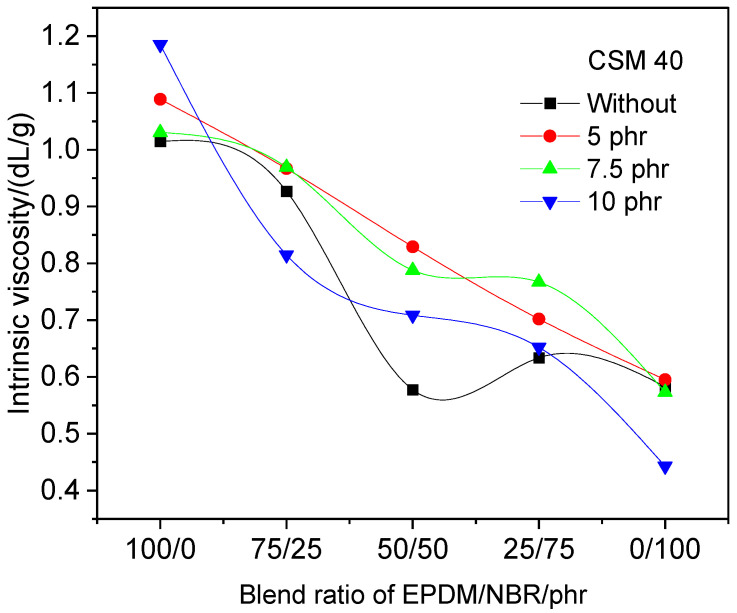
Intrinsic viscosity vs. blend ratio of EPDM/NBR blends containing different amounts of CSM 40.

Viscosity is considered a sensitive tool in studying the compatibility of polymer blends [71,72]. Since intrinsic viscosity is a characteristic property of a specific polymer and, in turn, of a given phase, measuring intrinsic viscosity in dilute solutions was used to study the compatibility of EPDM/NBR blends. The intrinsic viscosity of EPDM/NBR blend solutions was determined and plotted against the blend composition for each blend series, as shown in Figure 1.

The graph shows that in the absence of CSM 40, the dependence of intrinsic viscosity on the blend ratio was nonlinear. Also, with the addition of 7.5 and 10.0 phr of chlorosulfonated polyethylene, the dependence showed some deviation from the straight line connecting the intrinsic viscosity of individual NBR and EPDM rubbers. However, with the addition of 5.0 phr of CSM 40, the dependence was linear. This linearity [71] indicates that chlorosulfonated polyethylene can be used as a compatibilizer for EPDM/NBR blends, and 5.0 phr CSM 40 is the optimal amount to obtain a compatible blend.

Table 1 shows the formulation for a rubber compound with and without a compatibilizer, in which the ratio of EPDM S 501A and NBR 2645 rubbers was varied.

### 3.2. Curing (Rheometric) Properties and Crosslinking Density

**Figure 2:**
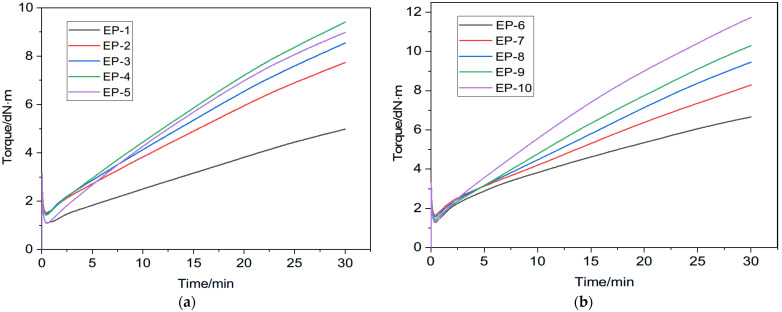
Curing curves for rubber compound at 150 °C: (**a**) (without compatibilizer), (**b**) (with compatibilizer).

Figure 2 shows the rheometric (curing) curves for various variants of the rubber compound with and without a compatibilizer at a temperature of 150 °C. Based on these curves, the vulcanization characteristics were determined and are presented in Table 2.

From the data in Table 2 it follows that an increase in the content of nitrile butadiene rubber NBR 2645 in the rubber compound (samples EP-1 ÷ EP-4) leads to an increase in the maximum *M*_H_ and minimum *M*_L_ torques and their difference (∆*M*). Whereas with a further increase in the content of NBR 2645 (samples EP-1 ÷ EP-5) a decrease in the scorch time and the optimum cure time of the rubber compound is observed, which is apparently associated with an increase in the proportion of double bonds of nitrile butadiene rubber in the elastomer matrix mixture, which are responsible for structuring the rubbers into a spatial vulcanization network. It is known [66,73,74,75,76,77,78,79] that the ∆*M* value is directly proportional to the chemical degree of crosslinking of the vulcanizate. In this regard, the EP-1 vulcanizate should have the lowest degree of crosslinking and, therefore, be characterized by lower strength indicators.

As can be seen from the data in Table 2, with an increase in the amount of NBR 2645 in the rubber compound including the compatibilizer chlorosulfonated polyethylene CSM 40, an increase in the maximum torque, a decrease (with the exception of EP-7 and EP-8) in the minimum torque, and an increase in their difference (∆*M*) are observed. At the same time, there is a decrease (except for EP-7) in the scorch time and a slight increase in the optimum cure time of the rubber compound. Based on the data of the ∆*M* indicator, it follows that the EP-6 vulcanizate should be characterized by the lowest degree of crosslinking, and the EP-10 vulcanizate by the highest degree of crosslinking. Consequently, the EP-6 vulcanized rubber should have lower strength properties, and the EP-10 vulcanizate should have higher strength properties.

Rubber compounds containing the compatibilizer are characterized by higher minimum torque values, which is due to increased compound viscosity, and higher maximum torque and ∆*M*, as well as shorter scorch time compared to the rubber compounds without the compatibilizer. The optimum cure time for both the compatibilizer-containing and non-compatibilizer-containing rubber compounds is virtually identical.

Table 3 shows the crosslinking density data for vulcanizates of rubber compounds that do not include and include CSM 40, respectively.

From the data in Table 3, it follows that the results of the study to determine the crosslinking density confirm the above assumption that the ∆*M* value is directly proportional to the crosslinking density ν_c_ of the vulcanizate. With an increase in the NBR 2645 content in the rubber compound, both without and with the compatibilizer, an increase in the value of ν_c_ of the vulcanizates is observed. As expected, among the rubber compounds without the compatibilizer, the vulcanizate EP-1 has the lowest ν_c_, and the vulcanizate EP-5 has the highest ν_c_. Among the vulcanizates, including the compatibilizer, the vulcanizate EP-6 has the lowest ν_c_, and the vulcanizate EP-10 has the highest ν_c_. It should be noted that vulcanizates with a compatibilizer are characterized by large ν_c_ values, in contrast to rubbers that do not contain a compatibilizer, which is presumably due to the additional structuring of chlorosulfonated polyethylene into a spatial network, caused by the interaction of chlorosulfone groups –SO_2_Cl with zinc oxide, which is part of the rubber compound.

### 3.3. Dynamic Mechanical Properties

Dynamic mechanical analysis is one of the important methods for studying the mechanical properties of polymeric materials depending on temperature, frequency and other factors, as well as for assessing the compatibility of polymer (rubber) phases.

**Figure 3:**
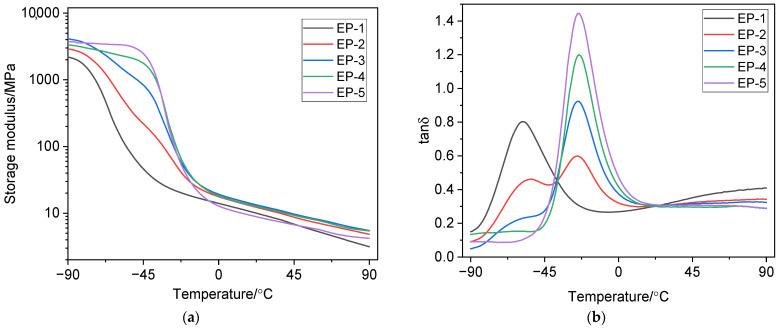
Temperature dependences of the storage modulus (**a**) and the tan delta (loss factor) (**b**) of vulcanizates without a compatibilizer.

**Figure 4:**
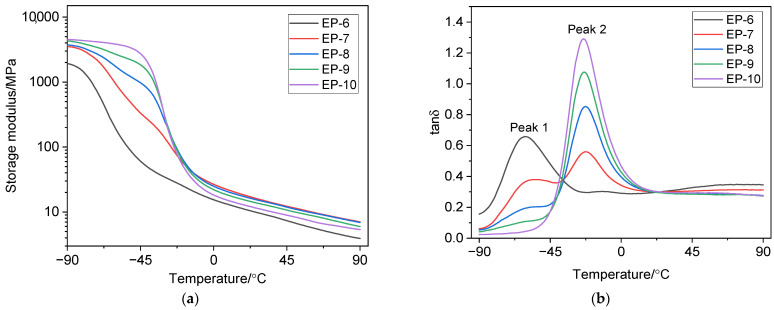
Temperature dependences of the storage modulus (**a**) and the tan delta (**b**) of vulcanizates with a compatibilizer.

The glass transition temperature *T*_g_ was determined as the temperature of the maximum in the temperature dependence of the loss factor *tan*δ. Figure 3 and Figure 4 show the temperature dependence curves of the storage modulus *E*′ and *tan*δ for the vulcanizates. Table 4 presents the dynamic parameters of the studied vulcanizates.

As Figure 3a and Figure 4a show, increasing the NBR 2645 content in the rubber compound increases the storage modulus of the vulcanizates in the glassy state. Moreover, vulcanizates EP-1 and EP-6 have the lowest *E*′ value, while vulcanizates EP-5 and EP-10 have the highest *E*′ value in the glassy state.

Figure 3b shows that the EP-1 vulcanizate exhibits a monomodal temperature dependence of *tan*δ. The peak at −58.0 °C (peak 1) with a *tan*δ_max_ value of 0.803 (Table 4) corresponds to the EPDM S 501A phase. The temperature dependence of *tan*δ for the EP-2 vulcanizate has a bimodal character, which indicates the existence of two separate peaks and, consequently, the incompatibility or segmental immiscibility of the polymer phases: the peak at −53.0 °C (peak 1) with the value of *tan*δ_max_ = 0.462 refers to the ethylene propylene diene monomer rubber phase, and the peak at −25.0 °C (peak 2) with the value of *tan*δ_max_ = 0.599 (Table 4)—to the NBR 2645 phase. The EP-3 vulcanizate is characterized by the presence of a shoulder peak on the temperature dependence of *tan*δ. For the EP-4 vulcanizate with a ratio of EPDM S 501A/NBR 2645 (25/75 phr), a monomodal type of temperature dependence *tan*δ is observed, which characterizes the improved compatibility of the rubber phases. Vulcanized rubber EP-5 also has a monomodal type of temperature dependence *tan*δ: the peak at −24.0 °C (peak 2) refers to the NBR 2645 phase.

A similar picture is observed for vulcanizates EP-6 ÷ EP-10 (Figure 4b): vulcanized rubbers EP-6 and EP-10 have one peak in the temperature dependence of *tan*δ. For vulcanizate EP-7, two glass transition temperatures associated with the EPDM S 501A and NBR 2645 phases are observed in the temperature dependence of *tan*δ. Vulcanizate EP-8 with a ratio of EPDM S 501A/NBR 2645 (50/50 phr) in the presence of 5 phr of compatibilizer CSM 40 is characterized by a shoulder peak. The presence of the shoulder indicates partial miscibility [80] of the EPDM and NBR phases at a given mass ratio.

The addition of a compatibilizer to the EPDM/NBR compound results in a slight shift in the *tan*δ peak toward lower temperatures, as well as a decrease in the height of the *tan*δ peak for vulcanized rubbers EP-7 and EP-8. For the vulcanizate EPDM S 501A/NBR 2645/CSM 40 at a ratio of 25/75/5 phr, a monomodal type of temperature dependence of *tan*δ is observed, which indicates improved phase interaction of rubbers.

It should be noted that the crosslinking density ν_c_ affects *tan*δ and chain mobility. This is because vulcanization, which involves crosslinking of rubber macromolecules, forms a spatial network that determines the mechanical properties of the vulcanized rubber. Chain mobility (segmental mobility) affects the mechanical losses that occur as a result of the translational movement of segments in a viscous medium. Vulcanizates with a compatibilizer, characterized by a higher ν_c_, have the lowest *tan*δ_max_ values compared to vulcanizates that do not contain chlorosulfonated polyethylene. Crosslinking density determines the density of the network structure. The denser the vulcanization network, the lower the chain mobility, which reduces *tan*δ.

The compatibility of EPDM and NBR blends with the addition of the compatibilizer CSM 40 is apparently ensured by the fact that the chlorine atoms in the chlorosulfonated polyethylene CSM 40 and the nitrogen atoms in the nitrile butadiene rubber participate in dipole–dipole interaction [81,82].

**Figure 5:**
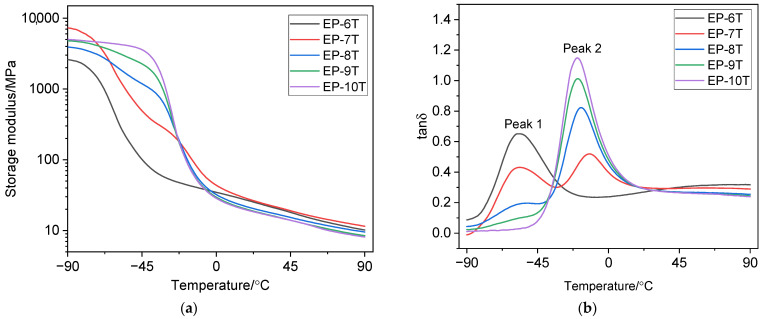
Temperature dependences of the storage modulus (**a**) and the tan delta (**b**) of vulcanizates with a compatibilizer after thermostatting.

We also studied the effect of thermostatting of vulcanizate samples containing a compatibilizer on the dynamic properties. The temperature dependence curves for *E*’ and *tan*δ for the samples after thermostatting are shown in Figure 5. The dynamic parameters of the vulcanizates after thermostatting are presented in Table 5.

As can be seen from Figure 5a, with an increase in the amount of NBR 2645 in the rubber compound, an increase in the *E*′ of the vulcanizates in the glassy state is observed. The EP-6T vulcanizate has the lowest *E*′ value, and the EP-7T vulcanizate has the highest *E*′ value in the glassy state. It can be noted that the nature of the temperature dependence curves of *tan*δ for vulcanizates with a compatibilizer after thermostatting remained virtually unchanged compared to the curves for vulcanized rubbers that were not subjected to thermostatting (Figure 5b). As can be seen from Figure 5b and from the data in Table 5, thermostatting of the vulcanizates is accompanied by a shift in the *tan*δ peak towards higher temperatures and a decrease in the height of the *tan*δ peak (except for peak 1, related to the EPDM phase, for vulcanized rubber EP-7T). This is probably explained by an increase in the efficiency and density of crosslinking after thermostatting of the vulcanizates.

### 3.4. Scanning Electron Microscopy (SEM)

**Figure 6:**
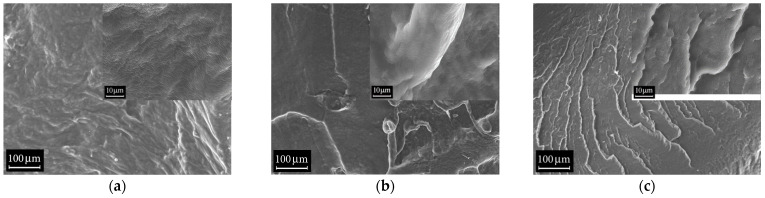
SEM images of the cleavage morphology of rubbers: (**a**) EPDM S 501A and (**b**) NBR 2645 and (**c**) compatibilizer CSM 40. Images were obtained at 300× magnification (insert 3K×) in SE2.

Both the original rubbers and their blends, as well as EPDM/NBR-based vulcanizates, were studied using the SEM method. The morphology of the fracture surfaces of EPDM S 501A, NBR 2645 rubbers and the compatibilizer CSM 40 is shown in Figure 6. It is evident that the morphology of the rubbers was slightly different: in the case of EPDM it was relatively smooth (Figure 6a), which at higher magnification (see inset) had a clearly defined folded structure; for NBR, in turn, many breaks and strands were observed (Figure 6b); small features of the morphology also had a folded structure, while in the case of the compatibilizer CSM 40 the morphology had a step-like appearance, characteristic of a fracture (Figure 6c); the fine fold structure was not observed and consisted of small particles. The nature of the fine folded structure was most likely due to the effect of the high-energy electron beam on the polymer base of the rubber (this effect was not studied in the present work).

**Figure 7:**
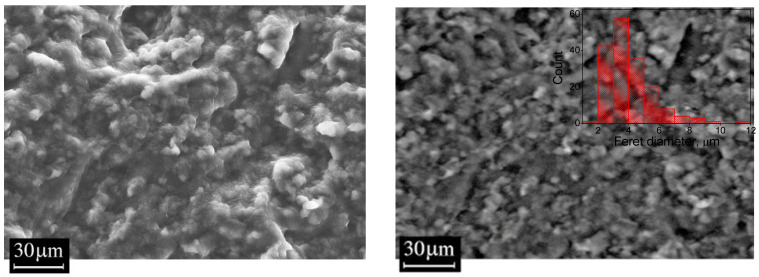
SEM images of an EPDM S 501A/NBR 2645 rubber blend with 5 phr of CSM 40 compatibilizer added. The images were obtained at 1K× magnification in SE2 (**left**) and BSE (**right**) modes. The inset shows the size distribution histogram for visually observable structural units.

Figure 6 shows cross-sectional images of a 50/50 phr EPDM S 501A/NBR 2645 rubber blend with 5 phr of CSM 40 compatibilizer added. Two images are presented, obtained with secondary (SE2) and backscattered (BSE) electron detectors. The first maximally displays the morphological contrast, the second the density contrast (Z-contrast). By comparing both contrasts, we can conclude that the blend consists of NBR granules embedded in an EPDM matrix. The characteristic granule size ranges from 2 to 10 µm (see the histogram in Figure 7). According to the results presented above, this case corresponds to partial miscibility of the rubbers.

**Figure 8:**
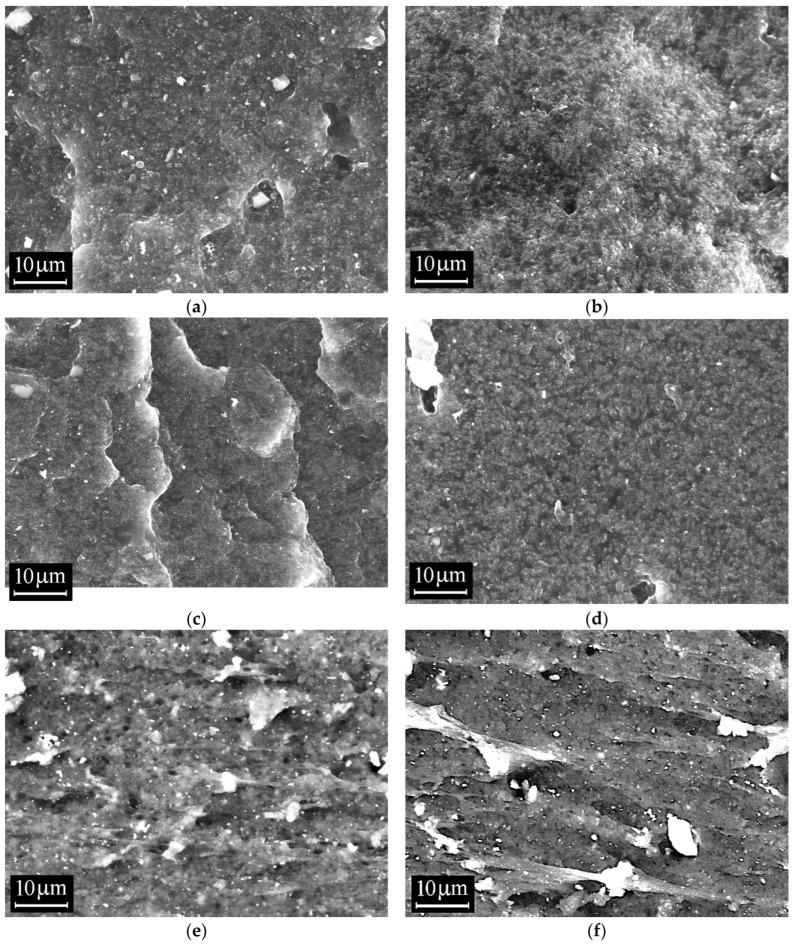

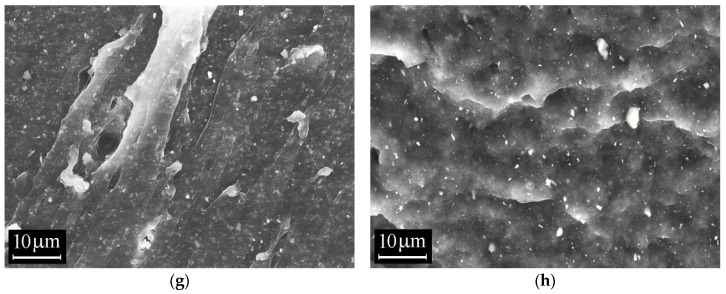
SEM images cross-section of rubber blends (3 kX, 20keV): (**a**) EP-1, (**c**) EP-3, (**e**) EP-4, (**g**) EP-5—without compatibilizer; (**b**) EP-6, (**d**) EP-8, (**f**) EP-9, (**h**) EP-10—with compatibilizer.

SEM images of cross-sections of the vulcanized rubbers studied are shown in Figure 8. On the left are SEM images of rubbers based on EPDM/NBR blends without a compatibilizer, and on the right are similar blends with the CSM 40 compatibilizer. Unlike the rubber blends, no structural features similar to those seen in Figure 7 (NBR granules) are observed. However, all vulcanizate samples exhibit a contrast in the smaller granules, the characteristic sizes of which, according to an assessment performed using ImageJ 1.54d software tools, range from 40 to 120 nm with a peak near 80 nm. All these factors indicate that the observed contrast is due to the carbon filler, which, on the one hand, is of no interest for further analysis, and on the other, interferes with the detection of other features, if any.

Comparing the images of chips with and without the compatibilizer, it can be seen that for vulcanizate samples EPDM/NBR at ratios of 100/0, 50/50, and 0/100 phr, the presence of CSM 40 leads to a slight smoothing of the chip morphology. A chip of the vulcanized rubber sample EPDM/NBR at 25/75 phr without the compatibilizer shows a pronounced microporous structure. With the addition of CSM 40, this feature is retained, but becomes less pronounced.

### 3.5. Physico-Mechanical Properties

**Figure 9:**
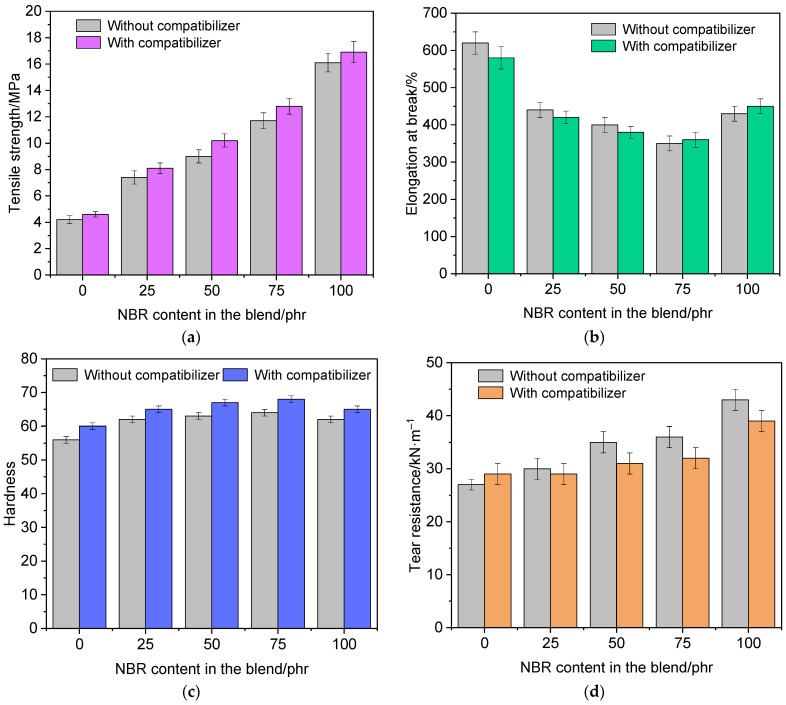

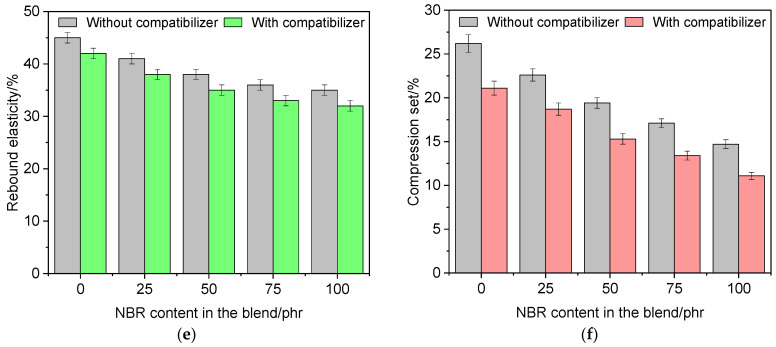
Physico-mechanical properties vulcanizates of different types of rubber compound: tensile strength (**a**), elongation at break (**b**), hardness (**c**), tear resistance (**d**), rebound elasticity (**e**) and compression set (**f**).

The results of the study of the physico-mechanical properties of vulcanizates of the studied rubber compound variants are presented in Figure 9.

As follows from Figure 9, an increase in the proportion of nitrile butadiene rubber NBR 2645 in the composition of the rubber compound, both without and with a compatibilizer, contributes to an increase in the tensile strength, hardness, tear resistance and a decrease in the elongation at break, rebound elasticity, and compression set of vulcanizates compared to the first version of vulcanized rubber EP-1, which does not include NBR 2645.

The introduction of a compatibilizer into the rubber compound helps to increase the conditional tensile strength by an average of 9.5%, hardness by 5.9% and reduce rebound elasticity by 8.4%, and compression set by 26.4%. According to the authors of [83], the magnitude of the compression set is greatly influenced by the size of the segment located between the nodes of the vulcanization network. With sufficiently long segments, i.e., with low cross-linking density, weak intermolecular bonds arise between the macromolecular chains. These bonds, under the influence of high temperatures and deformations, easily disintegrate and form in new locations, fixing the stress state, which negatively affects the level of residual compressive strain. The formation of a denser network of cross-links complicates the implementation of intermolecular physical interactions and, consequently, promotes a more complete recovery of the sample after the removal of the load. This assumption can probably explain the reduced level of accumulation of compression set in vulcanizates with a denser network of strong cross-links.

It should be noted that the increased values of the conditional tensile strength and hardness of vulcanizates with a compatibilizer compared to rubbers that do not contain CSM 40 are apparently due to the additional structuring of chlorosulfonated polyethylene into a spatial network and, as a consequence, higher values of the crosslinking density ν_c_ of the vulcanizates and ∆*M*.

Among rubbers based on EPDM and NBR, the vulcanizate EPDM S 501A/NBR 2645/CSM 40 (25/75/5 phr) has the highest tensile strength, hardness and the lowest rebound elasticity and compression set.

**Figure 10:**
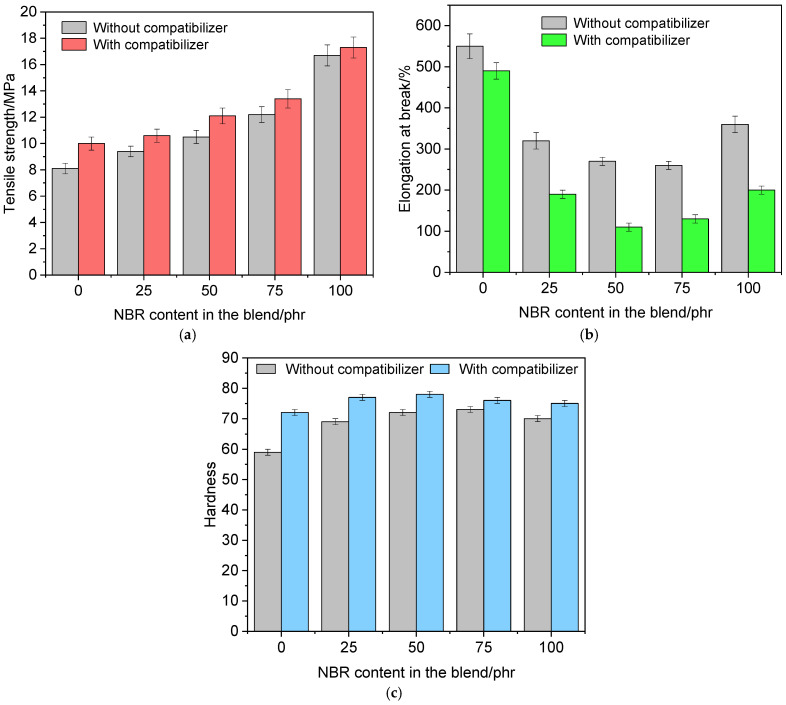
Indicators of tensile strength (**a**), elongation at break (**b**), and hardness (**c**) of vulcanized rubbers after thermostatting.

We further studied the elastic-strength properties of vulcanizates after thermostatting at 160 °C for 3 h, the results of which are presented in Figure 10.

As Figure 10 shows, thermostatting vulcanizates with and without a compatibilizer results in an increase in tensile strength and hardness and a decrease in elongation at break. For rubbers without a compatibilizer, an increase in tensile strength by 12.9% and hardness by 11.4% is observed, while for vulcanizates with a compatibilizer, an increase in tensile strength by 14.2% and hardness by 16.4% is observed. Thus, thermostatting vulcanizate samples at high temperatures leads to the formation of a denser network, which contributes to improved strength and hardness.

### 3.6. Performance Properties

Research into the performance properties of vulcanized rubbers is based on examining their resistance to aggressive environments. We examined changes in the elastic strength properties of vulcanizates after thermal aging in air at 100 °C for 24 h, as well as the mass of vulcanized rubber after 24 h of exposure to industrial oil I-20A and standard petroleum fluid SZhR-1 at room temperature. The change in mass (swelling) of vulcanized rubbers from the point of view of diffusion behavior is associated with the penetration of molecules of the liquid of the aggressive environment into the intermolecular spaces of the rubber and the weakening of its intermolecular bonds. The results are presented in Table 6.

From the data in Table 6 it follows that an increase in the amount of NBR 2645 in the rubber compound, both without and with a compatibilizer, leads to a decrease in changes in the elastic-strength properties and hardness of vulcanizates after daily thermal aging in air and their mass after exposure to industrial oil I-20A and SZhR-1. Among the vulcanizates based on EPDM and NBR, the vulcanizate of the rubber compound EP-9, which includes a compound of rubbers with a ratio of EPDM S 501A/NBR 2645 (25/75 phr) in the presence of 5 phr of the compatibilizer CSM 40, is characterized by the smallest changes in these properties. This is explained by the fact that this vulcanizate has the highest crosslinking density ν_c_ among vulcanized rubbers based on EPDM and NBR, which hinders the penetration (diffusion) of industrial oil I-20A and standard petroleum liquid SZhR-1 into the elastomer matrix.

## 4. Conclusions

The potential of using chlorosulfonated polyethylene CSM 40 as a compatibilizer for a binary blend of ethylene propylene diene monomer EPDM S 501A and nitrile butadiene NBR 2645 rubbers is demonstrated. Intrinsic viscosity measurements showed that CSM 40 at 5.0 phr improved the compatibility of the PDM/NBR blends. It was revealed that the rubber compound containing CSM 40 are characterized by higher values of the minimum and maximum torques and their difference, as well as a shorter scorch time compared to the rubber compound without the compatibilizer. Using the DMA method, it was established that the temperature dependence of *tan*δ for vulcanizates both containing and without a compatibilizer, based on a blend of EPDM S 501A/NBR 2645 rubbers at a ratio of 75/25 phr, has a bimodal character, indicating the incompatibility of the polymer phases. For EPDM S 501A/NBR 2645 (25/75 phr) vulcanizates with and without a compatibilizer, a monomodal temperature dependence of *tan*δ is observed, characterizing the improved compatibility of the rubber phases. It is shown that thermostatting vulcanizates containing a compatibilizer leads to a shift in the *tan*δ peak toward higher temperatures and a decrease in the height of the *tan*δ peak, apparently due to an increase in the efficiency and density of crosslinking after thermostatting. SEM of the vulcanizates showed that the EPDM/NBR sample with a mass ratio of 25/75 phr without a compatibilizer is characterized by a pronounced microporous structure, and with the addition of chlorosulfonated polyethylene, this feature is retained, but becomes less pronounced. It was found that increasing the NBR 2645 content in rubber compounds, both those containing and without a compatibilizer, leads to increased tensile strength, hardness, and tear resistance, and decreased elongation at break, rebound elasticity, and compression set of the vulcanizates compared to vulcanized rubber without NBR 2645. Vulcanizates containing CSM 40 exhibit enhanced strength and hardness compared to vulcanized rubber without a compatibilizer. Thermostatic curing of vulcanized rubbers with and without a compatibilizer increases tensile strength, hardness, and decreases elongation at break. The results of the study of performance properties showed that with an increase in the proportion of NBR 2645 in the rubber compound with and without a compatibilizer, a decrease in changes in the elastic-strength properties and hardness of vulcanizates after daily thermal aging in air and their mass after exposure to industrial oil I-20A and SZhR-1 is observed. It was established that the rubber composition based on EPDM/NBR (25/75 phr), containing the compatibilizer CSM 40 in the amount of 5.0 phr (2.88 wt.%), is characterized by stable physico-mechanical and improved performance properties, and can be used for the manufacture of rubber products operated under conditions of exposure to oils and hydrocarbon environments.

## Figures and Tables

**Table 1:** Formulation for a rubber compound with and without a compatibilizer.

Rubbers and Ingredients	Rubber Compound Options (phr *)									
	EP-1	EP-2	EP-3	EP-4	EP-5	EP-6	EP-7	EP-8	EP-9	EP-10
EPDM S 501A	100.0	75.0	50.0	25.0	0	100.0	75.0	50.0	25.0	0
NBR 2645	0	25.0	50.0	75.0	100.0	0	25.0	50.0	75.0	100.0
CSM 40	0	0	0	0	0	5.0	5.0	5.0	5.0	5.0
Luperox F 40P E	6.0	6.0	6.0	6.0	6.0	6.0	6.0	6.0	6.0	6.0
Triallyl cyanurate TAC 70	2.0	2.0	2.0	2.0	2.0	2.0	2.0	2.0	2.0	2.0
ZnO	5.0	5.0	5.0	5.0	5.0	5.0	5.0	5.0	5.0	5.0
Stearic acid	2.0	2.0	2.0	2.0	2.0	2.0	2.0	2.0	2.0	2.0
Antioxidant 445	1.5	1.5	1.5	1.5	1.5	1.5	1.5	1.5	1.5	1.5
Nickel dibutyldithiocarbamate	1.0	1.0	1.0	1.0	1.0	1.0	1.0	1.0	1.0	1.0
Wax ZV-P	1.0	1.0	1.0	1.0	1.0	1.0	1.0	1.0	1.0	1.0
Carbon black P 324	30.0	30.0	30.0	30.0	30.0	30.0	30.0	30.0	30.0	30.0
Carbon black P 514	15.0	15.0	15.0	15.0	15.0	15.0	15.0	15.0	15.0	15.0
Naphthenic oil NMR-12	5.0	5.0	5.0	5.0	5.0	5.0	5.0	5.0	5.0	5.0
Total weight	168.5	168.5	168.5	168.5	168.5	173.5	173.5	173.5	173.5	173.5

* Parts per hundred parts of rubber.

**Table 2:** Curing characteristics of rubber compound with and without a compatibilizer.

Characteristics	Samples									
	EP-1	EP-2	EP-3	EP-4	EP-5	EP-6	EP-7	EP-8	EP-9	EP-10
*M*_L_, dN·m	1.54	1.90	1.81	1.72	1.35	1.82	2.08	1.92	1.72	1.59
*M*_H_, dN·m	5.16	7.85	8.64	9.47	9.03	6.84	8.42	9.53	10.34	11.78
∆*M*, dN·m	3.62	5.95	6.83	7.75	7.68	5.02	6.34	7.61	8.62	10.19
*t*_s2_, min	14.55	8.54	7.34	6.53	6.21	7.05	7.39	6.49	5.80	4.27
*t*_90_, min	26.32	26.19	26.17	26.08	25.59	25.41	26.40	26.18	26.14	25.91

Notation: *M*_H_ is the maximum torque; *M*_L_ is the minimum torque; ∆*M* is the difference between maximum and minimum torque; *t*_s2_ is the scorch time; *t*_90_ is the optimum cure time.

**Table 3:** Crosslinking density of the studied vulcanizates.

Characteristics	Samples									
	EP-1	EP-2	EP-3	EP-4	EP-5	EP-6	EP-7	EP-8	EP-9	EP-10
$\rho_{r}$, g/cm^3^	0.860	0.885	0.910	0.935	0.960	0.872	0.896	0.920	0.944	0.968
χ	0.496	0.476	0.455	0.435	0.414	0.491	0.472	0.452	0.433	0.413
ν_r_	0.190 ± 0.001	0.289 ± 0.001	0.308 ± 0.001	0.318 ± 0.001	0.313 ± 0.001	0.215 ± 0.002	0.310 ± 0.001	0.330 ± 0.001	0.340 ± 0.001	0.339 ± 0.002
M_c_ × 10^−3^, g/mol	27.632 ± 0.463	3.942 ± 0.040	2.965 ± 0.027	2.509 ± 0.022	2.438 ± 0.021	10.415 ± 0.281	3.164 ± 0.030	2.420 ± 0.021	2.080 ± 0.017	1.972 ± 0.032
ν_c_ × 10^4^, mol/cm^3^	0.181 ± 0.003	1.268 ± 0.013	1.686 ± 0.015	1.993 ± 0.017	2.051 ± 0.018	0.480 ± 0.013	1.580 ± 0.015	2.066 ± 0.018	2.404 ± 0.020	2.535 ± 0.040

Notation: $\rho_{r}$ is the density of rubber; χ is the Flory-Huggins interaction parameter; ν_r_ is the volume fraction of rubber in the swollen vulcanizate; M_c_ is the average molecular weight of a segment of the molecular chain; ν_c_ is the crosslink density.

**Table 4:** Results of DMA of vulcanizates with and without compatibilizer.

Samples	Peak 1		Peak 2	
	*T*_g_, °C	*tan*δ_max_	*T*_g_, °C	*tan*δ_max_
EP-1	−58.0	0.803	–	–
EP-2	−53.0	0.462	−25.0	0.599
EP-3	shoulder		−25.0	0.924
EP-4	–	–	−24.0	1.200
EP-5	–	–	−24.0	1.445
EP-6	−61.0	0.658	–	–
EP-7	−55.0	0.380	−23.0	0.559
EP-8	shoulder		−23.0	0.853
EP-9	–	–	−24.0	1.075
EP-10	–	–	−24.0	1.291

**Table 5:** DMA of vulcanizates containing a compatibilizer after thermostatting.

Samples *	Peak 1		Peak 2	
	*T*_g_, °C	*tan*δ_max_	*T*_g_, °C	*tan*δ_max_
EP-6T	−57.0	0.652	–	–
EP-7T	−56.0	0.431	−12.0	0.520
EP-8T	shoulder		−18.0	0.822
EP-9T	–	–	−20.0	1.012
EP-10T	–	–	−20.0	1.149

* Samples of vulcanizates including a compatibilizer after thermostatting are marked as EP-6T ÷ EP-10T.

**Table 6:** Performance properties of vulcanizates with and without a compatibilizer.

Characteristics	Samples									
	EP-1	EP-2	EP-3	EP-4	EP-5	EP-6	EP-7	EP-8	EP-9	EP-10
Changes in the properties of vulcanizates after aging in air at 100 °C for 24 h										
Δ*f*_p_, %	+ (14.1 ± 0.7)	+ (13.2 ± 0.6)	+ (12.8 ± 0.6)	+ (12.1 ± 0.6)	+ (11.7 ± 0.5)	+ (17.2 ± 0.9)	+ (16.1 ± 0.8)	+ (11.6 ± 0.6)	+ (10.0 ± 0.5)	+ (10.8 ± 0.5)
Δ*ε_p_*, %	+ (16.1 ± 0.8)	+ (15.0 ± 0.7)	+ (12.5 ± 0.6)	− (11.4 ± 0.6)	− (15.3 ± 0.7)	+ (14.3 ± 0.7)	+ 13.9 ± 0.7	+ (12.7 ± 0.6)	- (8.3 ± 0.4)	− (12.2 ± 0.6)
Δ*H*, Shore A units	−(2 ± 1)	+(1 ± 1)	+(1 ± 1)	−(1 ± 1)	−(1 ± 1)	+(3 ± 1)	+(3 ± 1)	+(3 ± 1)	+(1 ± 1)	+(2 ± 1)
Change in the mass of vulcanizates after exposure to aggressive environments at 23 °C for 24 h										
Δ*m* (oil I-20A), %	32.04 ± 0.34	17.33 ± 0.31	9.65 ± 0.23	2.55 ± 0.12	0.18 ± 0.01	26.83 ± 0.59	16.42 ± 0.61	9.47 ± 0.13	2.48 ± 0.11	0.14 ± 0.01
Δ*m* (SZhR-1), %	8.75 ± 0.33	5.96 ± 0.11	3.41 ± 0.12	0.72 ± 0.03	−(0.06 ± 0.01)	8.46 ± 0.19	5.68 ± 0.09	3.32 ± 0.10	0.65 ± 0.02	−(0.04 ± 0.01)

Notation: ∆*f*_p_, ∆*ε_p_*, Δ*m*—relative change in tensile strength, elongation at break and mass; ∆*H*—difference in rubber hardness before and after exposure to an aggressive environment.

## Data Availability

The original contributions presented in this study are included in the article. Further inquiries can be directed to the corresponding author E.E.
